# Linking Endotoxins, African Dust PM_10_ and Asthma in an Urban and Rural Environment of Puerto Rico

**DOI:** 10.1155/2015/784212

**Published:** 2015-11-22

**Authors:** Mario G. Ortiz-Martínez, Rosa I. Rodríguez-Cotto, Mónica A. Ortiz-Rivera, Cedric W. Pluguez-Turull, Braulio D. Jiménez-Vélez

**Affiliations:** ^1^Department of Biochemistry, University of Puerto Rico-Medical Sciences Campus, San Juan, PR 00935, USA; ^2^Center for Environmental and Toxicological Research, University of Puerto Rico-Medical Sciences Campus, San Juan, PR 00935, USA; ^3^Department of Biology, University of Puerto Rico at Humacao, Humacao, PR 00792, USA; ^4^School of Medicine, University of Puerto Rico-Medical Sciences Campus, San Juan, PR 00931, USA; ^5^Department of Radiology, University of Texas Health Science Center at San Antonio (UTHSCSA), San Antonio, TX 78229, USA

## Abstract

African Dust Events (ADE) are a seasonal phenomenon that has been suggested to exacerbate respiratory and proinflammatory diseases in Puerto Rico (PR). Increases in PM_10_ concentration and the effects of biological endotoxins (ENX) are critical factors to consider during these storms. ENX promote proinflammatory responses in lungs of susceptible individuals through activation of the Toll-like receptors (TLR2/4) signaling pathways. The objective of the study was to evaluate the toxicological and proinflammatory responses stimulated by ADE PM_10_ ENX reaching PR using human bronchial epithelial cells. PM_10_ organic extracts from a rural and urban site in PR (March 2004) were obtained from ADE and non-ADE and compared. A retrospective data analysis (PM_10_ concentration, aerosol images, and pediatric asthma claims) was performed from 2000 to 2012 with particular emphasis in 2004 to classify PM samples. Urban extracts were highly toxic, proinflammatory (IL-6/IL-8 secretion), and induced higher TLR4 expression and NF-*κ*B activation compared to rural extracts. ENX were found to contribute to cytotoxicity and inflammatory responses provoked by urban ADE PM_10_ exposure suggesting a synergistic potency of local and natural ENX incoming from ADE. The contribution of ADE PM_10_ ENX is valuable in order to understand interactions and action mechanisms of airborne pollutants as asthma triggers in PR.

## 1. Introduction

Puerto Rico receives the impact of the global phenomenon known as African Dust Events (ADE)or storms [1–3]. This atmospheric formation is one of the largest in the world capable of transporting billion of tons of mineral dust from the northwest African region of Sahara-Sahel to the Caribbean and the Americas. It also contributes to dust transport into the south region of Europe and to the east coast of Asia [4]. Dust particles comprise a wide range of components derived from biogenic materials such as pollen, microorganisms, insects (e.g., locusts); fossil fuel, biomass burning and mostly eroded mineral soils [1, 4, 5]. PM_10_ particle pollution is mainly derived from anthropogenic and natural sources such as ADE influenced by input of warm air masses; and traffic and industry emissions from urban environments and secondary atmospheric processes [6–8].

It has been reported that approximately half of the bacterial population isolated from ADE particles are Gram-negative containing ENX, also known as Lipopolysaccharide (LPS) [1, 9]. An estimate of ~10^9^ bacteria is found per gram of top soil; thus ADE can mobilize high microbial masses downwind; and given that dust episodes are common, high incidences of asthma related to LPS may be encountered [1, 9]. ENX levels present in PM_10_ (whole extracts) collected throughout the Atlantic Ocean were reported as high as 6.41 EU/m^3^ during an ADE compared to 0.50 EU/m^3^ and 0.80 EU/m^3^ before and after the respective ADE [3]. ADE PM_10_ pollution and the high ENX concentration found in particles suggests that it could very well be an important PM biological component influencing respiratory and cardiovascular diseases [3, 5, 10, 11]. In fact, the United States Environmental Protection Agency (2009) stated that there was suggestive evidence about a causal relationship between short-term coarse particle exposure and cardiovascular/respiratory/mortality effects [12]. Moreover, PM_10_ concentration can reach high levels (up to 263 *µ*g/m^3^) during dust storm days and be associated to increased mortality compared to clear days (up to 42 *µ*g/m^3^), increased respiratory hospital admissions, and outpatient visits for bronchitis, asthma, and upper respiratory tract infections [13].

Exposures to ENX along with other environmental agents such as indoor allergens, viruses, and air pollutants (e.g., particulate matter or PM) have been linked to worsened lung function, atopy, enhancement of asthma pathogenesis, and/or already established inflammation [14, 15]. These molecules have also been associated with the biological action (e.g., inflammatory effects) of ambient particles in* in vitro* and* in vivo* models [3, 16–18].

The bronchial epithelium represents the first line of pulmonary defense, functioning as a physicochemical barrier to the external environment and initiating the required signals for systemic response [19, 20]. It is capable of recognizing microorganisms and their products, secreting antimicrobial factors and other molecules involved in inflammatory responses [19, 20]. ENX can stimulate the innate immune system, causing the production of inflammatory cytokines, maturation of antigen-presenting cells, and upregulation of receptors, and even can cause influx of macrophage like cells to the alveolar space, which can enhance inflammation in the asthmatic lung [21–23].

The discovery of pattern recognition receptors (capable of sensing pathogen associated molecular patterns or PAMPs), Toll-like receptors (TLRs) functional expression in respiratory airway epithelial cells, which are involved in allergic sensitization, underlines their importance in inflammation and the pathogenesis of respiratory disorders such as asthma and allergy [24–28]. These receptors have the capacity of signaling to secrete diffusible chemotactic molecules and cytokines, upregulate surface adhesion molecules, and enhance expression of antimicrobial peptides [29]. Overall, TLRs are involved in tissue repair and inflammation of the lung environment [30]. Eleven TLR family members have been identified in humans [20, 31]. Expression of TLR1–10 has been determined in primary as well as human bronchial epithelial cells, BEAS-2B [24, 30, 32], where TLR7, TLR8, and TLR10 are the least expressed [25]. ENX signal is recognized predominantly through interaction with TLR4. TLR2 can recognize some atypical LPS [33] as long as peptidoglycans or PGN and lipopeptides derived from Gram-positive bacteria and fungal molecules [34]. Both receptors activate cellular events that lead to the recruitment of specific adaptor molecules. TLR4 responds to LPS via interaction with accessory proteins such as CD14, a glycophosphatidyl inositol-anchored coreceptor, which can exist in soluble and membrane bound forms [29, 35]. Two signaling pathways have been described for TLR4: the myeloid differentiation factor 88, MyD88-dependent (also used by TLR2 heterodimerized with TLR1 and TLR6), and MyD88-independent [30]. Both pathways lead to the degradation of inhibitor of NF-*κ*B (I*κ*B) and transcription factor, NF-*κ*B translocation to the nucleus in order to induce the expression of proinflammatory and host defense genes [20, 30, 36]. Some of the proinflammatory mediators induced by the MyD88-dependent pathway are cytokines and chemokines (e.g., IL-6 and IL-8) [20, 24, 30, 37]. IL-6 can stimulate B cells and promote T cell activation, growth and differentiation and IL-8 is a chemokine responsible for neutrophil chemoattraction and activation [29, 38–40]. Ultimately, neutrophils induce goblet cells growth, mucus production, and alveolar wall damage.

Our laboratory has studied the toxic and proinflammatory effects of rural ADE aqueous and organic PM_10_ and PM_2.5_ PR extracts* in vitro* using human bronchial epithelial cells, BEAS-2B [2, 3, 41–43]. Efforts were mainly directed towards elucidating the contribution of trace elements to those responses. In addition, chemical characterization (trace elements quantification) of African dust storms (PM_2.5_) collected in the Aerosol and Oceanographic Science Expedition (AEROSE) during March 2004 was also evaluated [44]. Other investigators have also studied the contribution of airborne particulate matter to proinflammatory outcomes in that type of cells [45–48]. This study aims to study the toxicological and proinflammatory contribution of biological constituents (ENX) present in Puerto Rican ADE PM_10_ collected at a rural and urban environment* in vitro*. The BEAS-2B cell line was employed for that purpose since it is suitable and one of the models most affected by the interaction, deposition, and toxic effects of air pollution particles has an important role in surveillance being easily activated by superficial exposure to microbial factors and is the most promising model to assess respiratory sensitization of chemical compounds [49, 50]. Therefore, this work aims to characterize the PM_10_ carried by ADE to Puerto Rico during 2004. Then, particular emphasis is given to the study of the* in vitro* proinflammatory potential of natural ENX carried to the island. PM_10_ from a rural site is employed and the input of anthropogenic ENX added from local urban sources is specially considered throughout the analysis.

## 2. Methodology

### 2.1. Retrospective Analysis, Site, and PM Filter Selection

**Figure 1:**
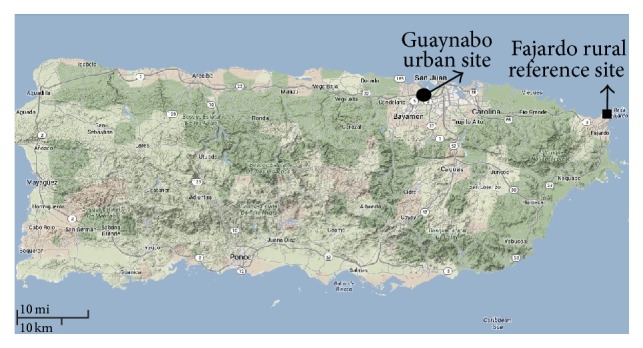
Map of Puerto Rico depicting the sites where PM_10_ was collected. The urban site, Guaynabo (Station #24: 18°:26:22/66°:06:54), is represented by a black dot and the rural reference site, Fajardo (Station #22: 18°:23:00/65°:37:10), by a black square. The figure was retrieved and modified from https://maps.google.com/.

ADE and non-ADE samples (PM_10_ quartz filters) were determined based on parameters and selection criteria previously reported [2, 3]. The PM filters were obtained from stations located at the municipalities of Fajardo/rural (reference) and Guaynabo/urban site (Figure 1). Briefly, PM_10_ data reported by the Puerto Rico Environmental Quality Board in conjunction with the aerosol index satellite image records (Total Ozone Mapping Spectrometer, TOMS) for each day was used to identify ADE and non-ADE days at a considerable confidence level [51]. All the dust events were classified and the mean duration through the year period of 2000–2012 was determined. An ADE day was defined as a day characterized by a PM_10_ concentration above the background level of 18.3 *µ*g/m^3^ with a concurrent presence of aerosol clouds over PR (as showed by TOMS). A day before and one to two days after the event were also considered as ADE. ADE input was analyzed to assess the frequency event arrival pattern to PR.

In an effort to study a possible correlation between ADE and respiratory health outcomes in PR, pediatric hospitalization cases due to respiratory conditions (by means of total services offered to asthma cases) were retrieved from the Health Insurance Administration (ASES) of PR [2] and evaluated. Data was meticulously revised and individuals affected by asthma were considered for one service per day, allowing for an appropriate distribution of total and age-categorized cases for statistical analyses (see Section 2.8 ahead).

### 2.2. PM_10_ Organic Extract Preparation

Organic extracts from PM_10_ quartz filters classified as ADE and non-ADE from March 2004 were Soxhlet extracted following the procedure described in Rodríguez-Cotto et al. [3]. Briefly, filters were submitted to a 48 hr extraction period in a 1 : 1 hexane to acetone solution (Fisher, Waltham, MA); the resulting extracts were dried under a gentle nitrogen stream and dissolved in dimethylsulfoxide (DMSO, Sigma, St. Louis, MO). Two organic composite samples at a final concentration of 100 mg/mL were prepared as stocks from each site corresponding to the same month. Both filters and extracts were stored at −20°C until further analyses.

### 2.3. Endotoxin Quantification

A 1/1000 dilution of the organic extracts was used to determine endotoxin concentrations employing the Kinetic Limulus Amebocyte Lysate Assay (*Kinetic-QCL*, Lonza, Walkersville, MD) as recommended by the manufacturer. The assay is based on the activation of a proteolytic cascade by ENX in the serum of the horseshoe crab* Limulus polyphemus*. A standard curve (50–0.005 EU/mL) was prepared from* Escherichia coli* O55:B5 endotoxin. Absorbance readings (405 nm) at 37°C were recorded using an EL_x_ 808 microplate reader (Biotek Instruments, Winooski, VT) and data analyzed using the WinKQCL Software (Lonza, Walkersville, MD). The detection limit for this assay is 0.005 EU/mL. Final results were expressed as EU/mg of extracted PM_10_.

### 2.4. Cell Exposures

Human bronchial epithelial cells (BEAS-2B, ATCC # CRL-9609) were cultured and maintained with Keratinocyte Growth Medium (KGM-2, Walkersville, MD) at 37°C, 5% CO_2_. Cells from passages 44–69 were grown in 96-well plates and exposed for 24 hr to different ADE/non-ADE PM_10_ organic (5–10 *µ*g/mL range) extract concentrations in three independent experiments. Negative controls were verified in three different ways: unexposed (media alone), dilution (vehicle: DMSO used in the organic extracts), and process (organic extract derived from a blank filter). Endotoxin inhibition experiments were conducted by preincubating the extracts to 10 *µ*g/mL Polymyxin B sulfate (PMB, Sigma, St. Louis, MO) in a sonic water bath for 30 min prior to cell treatment in order to prevent any aggregates and allow time for inhibition. Other negative and positive controls were exposed to media preincubated with PMB, LPS (0111:B4, Sigma, St. Louis, MO) and LPS pretreated with PMB. Cell supernatants were collected after each exposure and stored at −80°C until further cytokine analyses.

### 2.5. Cytotoxicity and Cytokine Determination

Cytotoxic effects of PM_10_ on exposed cells were assessed using the neutral red bioassay (Sigma, St. Louis, MO) as described elsewhere [2, 3]. After exposures, media were removed and neutral red dye was added at 100 *µ*g/mL for 3 hr. Following this incubation, cells were fixed with 1% calcium chloride (Sigma, St. Louis, MO) and 0.5% formaldehyde (Invitrogen, Life Technologies, Grand Island, NY), rinsed with phosphate buffered saline, and solubilized with 1% acetic acid (Fisher, Waltham, MA) and 50% ethanol. Cell viability was determined by absorbance at 540 nm in an Ultramark microplate reader (Bio-Rad, Hercules, CA). Eighty percent viability was considered as a threshold for cytotoxicity. Triton X-100 (Fisher, Walthman, MA) at 25 *µ*g/mL was used as a positive control for cytotoxicity. The levels of proinflammatory cytokines (IL-6 and IL-8) in cell supernatants of three independent experiments were simultaneously determined in cell supernatants using a multiplex bead assay (Millipore) in the dual laser flow analyzer with a Luminex 200 software (Luminex Corp., Austin, TX). Basically, multiplex bead assays are solid phase immunoassays that allow simultaneous quantitative detection theoretically of up to 100 analytes in a single microtiter by flow cytometry. The median fluorescence intensities of fluorochrome-conjugated antibodies bound to individual microspheres are derived from the flow analysis of 100 microspheres per well (in duplicate). Cytokine standard curves were constructed using a 5-parameter logistic fit [2, 3].

### 2.6. RNA Extraction and Quantitative Real-Time Polymerase Chain Reaction (PCR)

BEAS-2B cells were seeded in 96-well plates and exposed to ADE/Non-ADE PM_10_ extracts (Fajardo and Guaynabo) with and without the ENX inhibitor (PMB). The mRNA expression of TLRs and coreceptor CD14 were determined using quantitative Real-Time PCR at exposure times of 12 hr (TLR2) and 7 hr (TLR4), specific time points selected based on previous time course data which revealed peak expression of the receptors. Total RNA was isolated using Trizol Reagent (Invitrogen, Life Technologies, Grand Island, NY) and its integrity and purity were verified by gel electrophoresis. The cDNA was synthesized using High Capacity cDNA Reverse Transcription Kit (Applied Biosystems, Foster City, CA) and quantitation of 100 *µ*g/mL samples performed using a StepOne PCR instrument (Applied Biosystems, Foster City, CA) based on commercial Taqman assays and Gene Expression Master Mix (Applied Biosystems, Foster City, CA). The assays (probes and primers) were validated following the company's recommendations. Three to four independent experiments were run. The PCR program used consisted of 2 min at 50°C, 10 min at 95°C followed by 45 cycles of 15 s at 95°C, and 1 min at 60°C. Ct data was analyzed employing the StepOne software (Applied Biosystems, Foster City, CA) and normalized to the glyceraldehyde-3-phosphate dehydrogenase (GAPDH) housekeeping gene.

### 2.7. Protein Extraction and NF-*κ*B Activation Assay

BEAS-2B cells were seeded in 6-well plates and exposed to ADE/non-ADE PM_10_ organic extracts (Fajardo and Guaynabo) with and without PMB (ENX inhibitor) and incubated for 4 hr. Nuclear extracts were prepared using the Nuclear Extract Kit (Active Motif, Carlsbad, CA) following the manufacturer's guidelines. Protein concentrations were determined using the Bradford-based assay (Protein Assay, Bio-Rad, Hercules, CA) by measuring absorbance at 595 nm in an Ultramark microplate reader (Bio-Rad, Hercules, CA). Nuclear extracts (5 *µ*g protein) were evaluated in three to four independent experiments using the TransAM NF-*κ*B Enzyme-Linked Immunosorbent assay (ELISA) based kit (Active Motif, Carlsbad, CA) for NF-*κ*B activation. The kit functions through the use of oligonucleotides containing the NF-*κ*B consensus binding site which are immobilized in a 96-well plate and the range of detection is 0.2–10 *µ*g of nuclear extract. Absorbance was measured on a spectrophotometer (Bio-Rad, Hercules, CA) at 450 nm.

### 2.8. Statistical Analyses

Individual group differences were evaluated using the Student's *t* Test. Statistical significance was set at *p* ≤ 0.05 and results were graphed using the GraphPad InStat 3 software. A possible association between the pediatric asthma cases (total and subdivided in age groups) and three variables was tested through examining proportion differences and population homogeneity followed by a correlation analysis. The variables were (1) PM_10_ concentration; (2) PM_10_ increment (calculated from subtraction: average high concentration values − average low concentration values); and (3) ADE days reported for the rural (Fajardo) and urban (San Juan) site during 2004 and 2005 for comparison.

## 3. Results

### 3.1. Retrospective Analysis of ADE Duration, PM, and Asthma Cases

**Figure 2:**
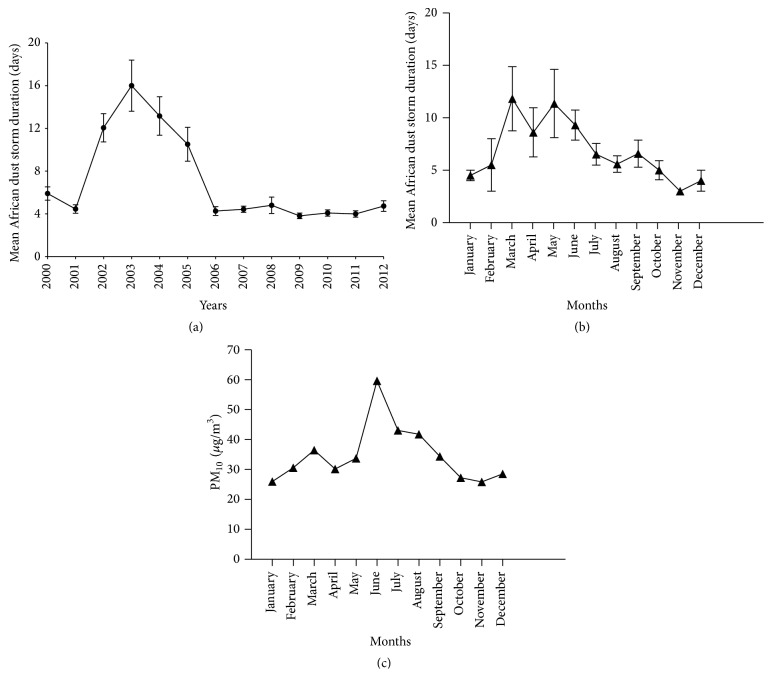
(a) Mean African Dust Storm duration in days for the period from 2000 to 2012; (b) Monthly mean (January to December) of African Dust Storm duration calculated for the entire period from 2000 to 2012; and (c) mean PM_10_ concentration (*µ*g/m^3^) in the urban site (Guaynabo, PR) during the representative year of 2004. Particulate matter from this year was used to perform the* in vitro* experiments.

The analysis of PM_10_ concentration data for a period of 12 years in conjunction with satellite imaging indicated that the mean ADE duration increased from 2001 until a peak in 2003 and 2004, decreasing to its background levels in 2006 followed by fairly constant mean values until 2012 (Figure 2(a)). When the average storm durations of all these years are analyzed in a per month basis a remarkable finding reveals that March is characterized by the longest ADE (11.8 days), followed by May (11.4 days), June (9.3 days), and September (5 days). Clearly, ADE duration varies throughout the year with a peak in March and decreasing after June (Figure 2(b)). The mean PM_10_ concentration for the urban site during 2004 also showed increase during the months of March and June (Figure 2(c)).

**Figure 3:**
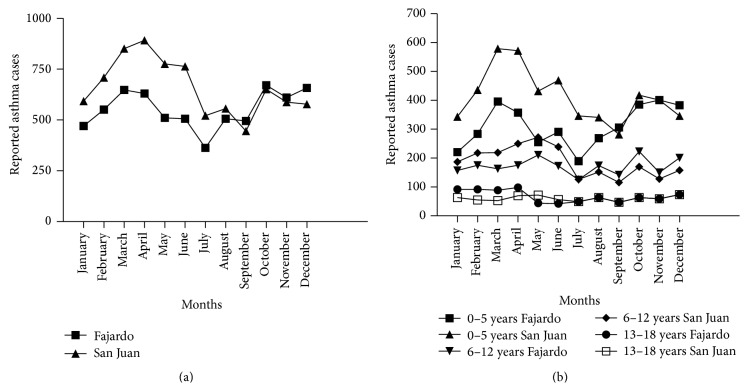
Annual Distribution of Pediatric Asthma Cases reported by the Health Insurance Administration of Puerto Rico (ASES) for Fajardo (rural) and San Juan (urban), Puerto Rico, during the year 2004. (a) Total and (b) categorized by age ranges: 0–5, 6–12, and 13–18 years. Asthma cases represent hospital emergency visits.

The asthma cases maintain a similar pattern (urban and rural) throughout the island for this year with the highest incidence occurring during the months of March 2004 (Figure 3(a)). A reevaluation of pediatric claims for 2004 shows that March has the highest number of asthma cases with the most significant affected category being children between 0 and 5 years of age, followed by those of 6–12 and 13–18 (Figure 3(b)). The number of 0–5 years cases for San Juan was significantly higher than those for Fajardo; however that was not observed for the 6–12 and 13–18 age ranges (*Z* = −6.09; *p* value = 0.00 for 0–5 years cases/*Z* = 4.83; *p* value = 1.00 for 6–12 years cases/*Z* = 2.56; *p* value = 0.995). A statistical analysis of the annual distribution of asthma cases during 2004 at the rural and urban sites revealed that case frequency distribution was different in all the 2004 months (*χ* ^2^ = 82.84 and 56.75; *p* value = 0.00). A correlation analysis revealed a relation between total asthma cases and both PM_10_ concentration (strong association with a correlation coefficient, *R* = 0.715 and *p* value = 0.009) and increment in the rural site, Fajardo, PR (Table 1). The PM_10_ concentration was related to asthma cases in the rural site distributed in all the 3 categories, the 0–5 years being the strongest one, but PM_10_ increment was only significantly correlated to that same category. Asthma cases during ADE days were also associated to the rural site (*R* = 0.548; *p* value = 0.042) and somewhat marginally to the urban site, San Juan, PR (*R* = 0.470; *p* value = 0.089). Adjustments made by location and year of event (2004 versus 2005) did not exhibit any significant correlations (*data not shown*).

### 3.2. Dose Response; Concentration and Inhibition of Endotoxins; and Cytokine Secretion

**Figure 4:**
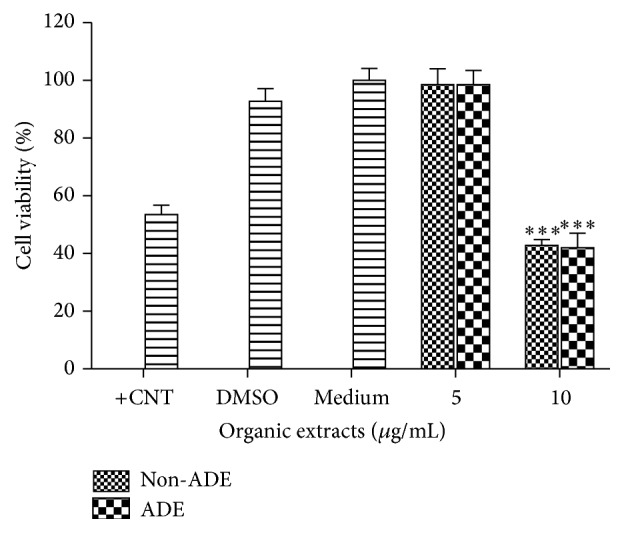
Cytotoxicity of BEAS-2B exposed to ADE/non-ADE PM_10_ organic extracts from the urban site. A value <80% was considered cytotoxic. Triton-X 25 *µ*g/mL was used as positive control (+CNT). Bars represent mean cell viability ± SEM, ^*∗∗∗*^ *p* < 0.001 of three independent experiments. Negative controls are medium and the carrier dimethylsulfoxide (DMSO) at 0.1%. Individual asterisks represent statistical significance compared to DMSO.

Urban and rural organic extracts were nontoxic at 5 *µ*g/mL and toxic at 10 *µ*g/mL (Figure 4). Concurrent experiments ran with organic extracts from a blank filter tested negative for cytotoxicity. The positive control, Lipopolysaccharide (LPS), was tested at 10 *µ*g/mL and was also nontoxic (*data not shown*).

**Figure 5:**
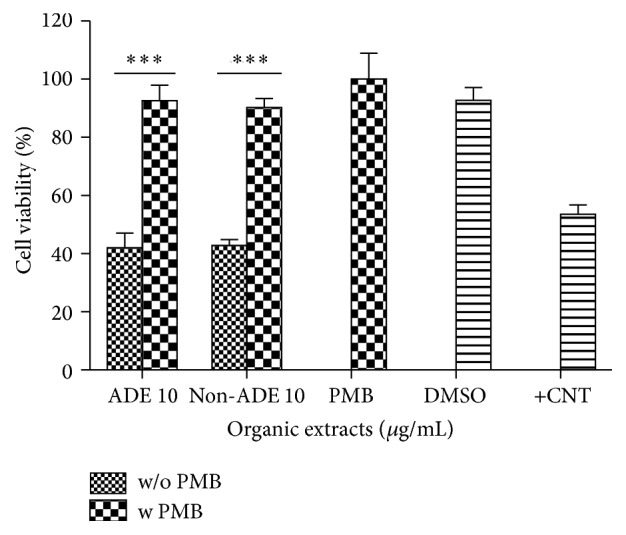
Cytotoxicity of BEAS-2B exposed to ADE/non-ADE PM_10_ organic extracts from the urban site after ENX inhibition with Polymyxin B (PMB). A value <80% was considered cytotoxic. The positive control (+CNT) of toxicity and negative controls are DMSO at 0.1% and PMB at 10 *µ*g/mL. Bars represent mean cell viability ± SEM, ^*∗∗∗*^ *p* < 0.001 of three independent experiments. Asterisks above line represent statistical significance between adjacent columns.

A concentration of 168 EU/mg was measured in the PM_10_ ADE sample from the urban compared to 116 in the non-ADE counterpart (Table 2). Endotoxin concentration in the non-ADE extract was comparable between the rural and urban sites but higher in the ADE extract from the urban site. PMB pretreatment to the extract reduced significantly (~45–50%) the inherent toxicity of the urban site both derived from ADE and non-ADE material (Figure 5). Endotoxins were present in a considerable level and responsible for most of the toxicity at the urban site.

**Figure 6:**
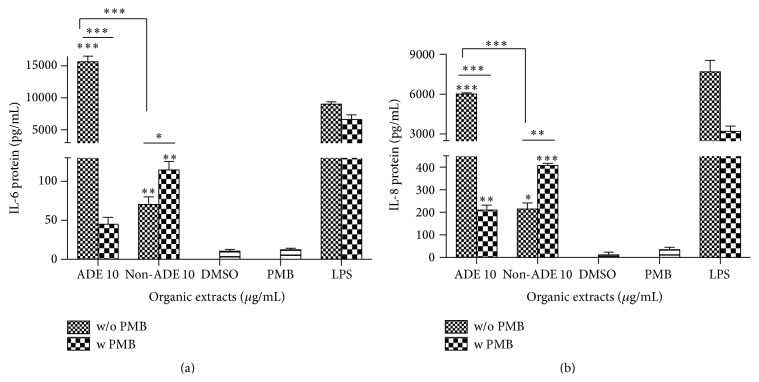
Induction of (a) IL-6 and (b) IL-8 in BEAS-2B exposed to PM_10_ organic extracts from the urban site. PMB stands for Polymyxin B sulfate at 10 *µ*g/mL. Lipopolysaccharide (LPS) at 10 *µ*g/mL was used as a positive control. Bars represent mean interleukin protein ± SEM, ^*∗∗∗*^ *p* < 0.001; ^*∗∗*^ *p* < 0.01; ^*∗*^ *p* < 0.05 of three independent experiments. Individual asterisks represent statistical significance compared to DMSO and those over line represent significance between adjacent columns.

Urban ADE extract is a strong inducer of IL-6 secretion in BEAS-2B lung epithelial cells resulting in 200-fold higher than the non-ADE extract (Figure 6(a)) while IL-8 was around 30-fold (Figure 6(b)). ENX inhibition was able to significantly decrease both interleukin levels induced by the urban ADE organic extract. Although IL-6 and IL-8 were also found to increase in the non-ADE extract at the urban site, the effect was significantly lower than that obtained from extracts during ADE (Figure 6(b)).

### 3.3. Gene Expression of Toll-Like Receptors and NF-*κ*B Activation

**Figure 7:**
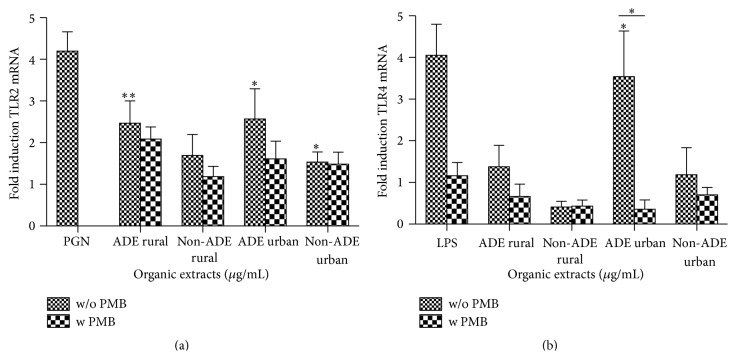
Gene expression of (a) Toll-like receptor 2 (TLR2) and (b) Toll-like receptor 4 (TLR4) in BEAS-2B exposed for 12 and 7 hours, respectively, to ADE/non-ADE PM_10_ organic extracts from the rural and urban sites at 10 *µ*g/mL and 5 *µ*g/mL, respectively, with and without ENX inhibition by Polymyxin B (PMB) at 10 *µ*g/mL. Peptidoglycans (PGN) at 100 *µ*g/mL and LPS at 10 *µ*g/mL were used as positive controls for TLR2 and TLR4, respectively. Induction of TLRs was normalized to GAPDH expression. Bars represent mean mRNA fold induction ± SEM, ^*∗*^ *p* < 0.05; ^*∗∗*^ *p* < 0.01 of three to four independent experiments. Individual asterisks represent statistical significance compared to DMSO and those over line significance between adjacent columns.

PM_10_ organic extracts were capable of inducing the mRNA levels of TLR2 and TLR4 at both the urban and rural sites. All ADE extracts induced TLR expression with the exception of TLR4 by the rural extract (Figure 7). However, the ADE urban extract had a significant influence on the expression of both TLR2 and TLR4 (Figures 7(a) and 7(b)). Neither of the non-ADE extracts was able to induce any TLR expression (Figure 7(a)). The use of PMB was not very effective in reducing TLR expression in non-ADE samples at either site. Conversely, the use of ENX inhibitor was effective in reducing only the TLR4 expression induced by the ADE extract from the urban site (Figure 7(b)). The ENX found in the urban ADE extract have the greatest effects on TLR2 (although not significant) and on TLR4 (3-fold) mRNA inductions. CD14 mRNA expression was not detected in BEAS-2B even in the positive control (LPS) treated samples.

**Figure 8:**
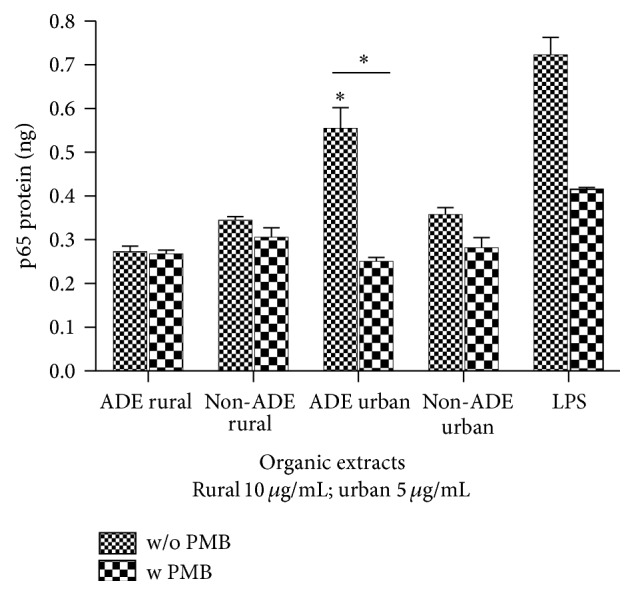
Nuclear factor kappa B (NF-*κ*B) activation measured in BEAS-2B exposed for 4 hrs to ADE/non-ADE PM_10_ organic extracts from the rural and urban sites at 10 *µ*g/mL and 5 *µ*g/mL, respectively, with and without ENX inhibition by Polymyxin B (PMB) at 10 *µ*g/mL. Bars represent mean transcription factor activation ± SEM, ^*∗*^ *p* < 0.05 of three to four independent experiments. Individual asterisks represent statistical significance compared to DMSO and those over line significance between adjacent columns.

Cells exposed to ADE/non-ADE organic extracts from both urban and rural sites showed nuclear factor kappa B (NF-*κ*B) activation at 4 hr (Figure 8) only for the ADE urban extract. The use of the endotoxin inhibitor, PMB, significantly reduced that activation to basal levels.

## 4. Discussion

High African dust (based on ground PM_10_ concentration and aerosol images) was reported for 2004 and specifically for the month of March and June [2, 3, 41]. The analysis of mean ADE duration per year and monthly mean from 2000–2012, compared to PM_10_ concentration for the urban site, undoubtedly supports the use of PM_10_ derived from March 2004. This particular year was one of the few with the most extended ADE and March was characterized by elevated mean ADE duration during the entire period added to an increase in PM_10_ concentration. This is the first time that an analysis for ADE duration in Puerto Rico has been reported. Furthermore, analysis of asthmatic data also showed an increase in pediatric cases during March, but this was not the case for June, even though it was a month with high PM_10_ concentration. This is consistent with studies from Montealegre et al. [52] in PR showing a lower occurrence of asthma attacks during June. The difference observed between March and June could be explained by various factors such as the difference between ADE occurrence, stability and composition, the length of cloud exposure days, and individual protection during a known dust season. In addition, it was found that asthma claims correlate to average PM_10_ concentration (in all three age categories), PM_10_ increment, and ADE days in 2004 in the rural site. The urban site extract showed a marginally significant correlation between asthma cases and ADE days. These associations are in agreement with what was reported for the Caribbean island of Trinidad back in 2001-2002 [53]. Pediatric asthma admissions were associated to increase dust cover during the year. The pediatric asthma cases reported at the urban site were significantly higher compared to the rural site, suggesting that urban activities are contributing to the exacerbation of this illness. Local air pollution at the urban site sets the conditions that are accentuated during the advent of African Dust Storms. Evidence of this is stated in a case-control study conducted by Loyo-Berríos et al. [54] which discovered that the risk of asthma attacks in children from Cataño (close to San Juan), PR, was associated to residing in the proximity of anthropogenic air pollution sources. Others have suggested the influence of high traffic and industries in risk to pediatric asthma [55]. The high number of cases reported during the fall and winter months, characterized by reduction in ADE and lower PM_10_ mass concentration [2, 3], could be attributed to other biological or chemical components such as pollen, dust mites, and fungal spores, which increase with humidity changes [56]. Endotoxin concentrations have been reported during the warmer spring and summer seasons, where bacteria and plants develop more [1, 57, 58].

The biological constituents of endotoxins are more prevalent and exhibit greater biological responses in the coarse PM than fine and ultrafine [59]. The use of the ENX inhibitor (PMB) demonstrated the importance of endotoxins on lung cell toxicity to urban PM_10_ organic extracts. The effect was not observed in the rural extracts [3], indicating that ENX derived from urban local emissions are potent mediators of toxicity. Moreover, endotoxins found in both extracts (ADE/Non-ADE) were similarly capable of causing cytotoxicity to the level of reaching basal cell viability, indicating that the potency of ENX is dependent on type rather than quantity itself, which also explains the difference between rural and organic fractions. Investigators studying the effect of urban air PM have found cytotoxic effects caused by ENX (in PM_10-2.5_ fraction) and have suggested that the inhibitory action of PMB may be slightly nonspecific but nonetheless measure of the microbial contribution to cellular responses [59, 60]. The authors found an inhibition of cytotoxicity upon use of PMB in Gram-negative but not in Gram-positive bacteria; however in the case of inflammatory responses (which are discussed later) a higher reduction was observed with Gram-negative bacteria. Endotoxins appear to affect cellular responses irrespective of the content, which could explain the similar effect obtained between ADE/non-ADE extracts. These molecules are one of the most important and studied PM constituents to which many respiratory health problems (respiratory symptoms, atopic reactions, airway and systemic inflammation, and pulmonary function decline) have been attributed [14, 61]. Asthma is thought to worsen to increased ENX exposure, particularly in individuals sensitized against allergens. Also, these toxins can intensify existing sensitization, eosinophilic influx into lung tissue and mucus production, among others [14]. Previous reports have acknowledged the presence of Gram-negative bacteria in African Dust Storms, supporting the presence of ENX in transatlantic material [1, 9]. Endotoxins in ADE urban organic extracts were higher than in rural extracts (168 vs 114 EU/mg) reported by Rodríguez-Cotto et al. [3] (Table 2). There is conflicting evidence relating human activity and ENX in urban environments. Some state that human activity increases microbial matter (increasing aero-dispersed ENX) suspended in the atmosphere [61–63]. Others argue that ENX levels tend to be lower in urban settings [14, 64]. These reports suggest that ENX in air will depend on the characteristics of the environment. In the present work, the toxins appear higher in the Puerto Rican urban environment, which supports the hypothesis that human activity modifies the environment enhancing ENX enrichment. This local urban load is augmented by incoming contributions from African Dust, which are prolonged during March, April, and May months.

PM_10_ have been reported to induce the release of cytokines in BEAS-2B and alveolar macrophages [46, 65–67]. Our study reports that ADE PM_10_ organic extracts recovered in PR also induce the release of IL-6 and IL-8, illustrating their inflammatory strength on human bronchial epithelial cells. Urban extracts are particularly potent inducers. The release of IL-6 and IL-8 was partial but predominantly reduced upon PMB pretreatment demonstrating the importance of ENX involvement in this response. This effect has also been described after PMB pretreatment in PM_10_ using human alveolar and mouse macrophages [65, 68, 69]. In the case of human alveolar macrophages a dramatic IL-6 reduction of 59% was found upon air pollution (insoluble PM_10_ characterized by 4 EU/mL) treatment with PMB; and on the other side, mouse macrophages exposed to urban particles exhibited IL-6 reduction after PMB treatment. The proinflammatory effect of endotoxins in PM_10_ was also previously described in alveolar epithelial cells (A549) exposed to urban versus rural PM_10_, where even though rural particles were more IL-6 and IL-8 inducers, an association was observed with higher endotoxin content in the particles [70]. Other studies have indicated that endotoxin content is not directly correlated with inflammatory response [59, 60], giving importance to ENX types and other microbial and organic components in the extracts. The use of PMB increased the secretion of IL-6 and IL-8 for the non-ADE urban extract instead of reducing it. This could be due to differences in ENX types extracted and present during different times and locations. For example, it has been shown that lung cell responses vary depending on the* E. coli* serotype or bacteria species present. LPS from* P. aeruginosa* is potentially more inflammatory than LPS from* E. coli* [71]. It is also possible that other biological agents such as peptidoglycans and fungal spores, not inactivated by PMB, may contribute to IL release [18, 72]. The ENX may interact with some of those other agents, possibly preventing them to effectively induce the release of proinflammatory cytokines. Upon PMB pretreatment the effect is reversed and cytokine induction can take place. Differences in the contribution of ENX to the proinflammatory response could also be explained by distinct components adsorbed to PM and the particle sources [68]. Interactions between PM constituents and ENX have been proposed between, for example, ENX/metals and ENX/inactive fungi (e.g., *β*-glucans) [15, 65, 73].

ENX are recognized by TLRs (mostly TLR4 and partially TLR2) on the bronchial epithelial cell surface, activating intracellular signal transduction that will ultimately lead to the induction of proinflammatory genes such as IL-6 and IL-8 via the transcription factor, NF-*κ*B nuclear translocation. Both TLRs were found to be induced by organic extracts, but the TLR4 exhibited the stronger effect in BEAS-2B after exposure to the urban ADE extract. Pretreatment with PMB was able to reduce TLR4 expression, indicating the importance of ENX in positively regulating its own receptor expression. Biological constituents in PM have been recognized to be important agents in TLR4/2 signaling since heat inactivation of microbial materials is able to diminish TLR expression [72, 74]. The TLR2 was induced by both rural and urban ADE extracts showing cell responsiveness to other molecular patterns (e.g., other bacterial products: peptidoglycans and lipopeptides) recognized by TLR2 possibly present in ADE, despite the fact that TLR2 expression is lower in this cell line [75]. Moreover, this increase in expression was not due to atypical ENX as proven by the lack of response due to ENX inhibition (PMB). PM_10_ extracts are largely TLR4-dependent and activate alveolar macrophages to produce cytokines while PM_2.5_ tends to be TLR2-dependent and activates bronchial epithelial cells for the same outcome [74]. Reinforcing these distinct findings, expression of both TLR2 and TLR4 mRNAs has been reported by others in BEAS-2B [25, 76] and TLR2 expression has been found to be ligand-dependent [76]. Overall, we found that PM_10_ extracts induce TLR2 expression in rural and urban environments while TLR4 expression is only induced by urban dust. Other organic constituents are responsible for the inflammatory responses induced by ADE rural exposure. Expression of these TLRs in bronchial epithelial cells supports the triggering of innate immunity in the respiratory epithelium.

Expression of CD14 was not detected in BEAS-2B stimulated with LPS. Contradictory results have been shown by different studies in terms of its mRNA expression (low levels or none) in BEAS-2B [77, 78]. A CD14-independent mechanism of cell activation has been suggested as possible in bronchial epithelial cells [26, 35]. These differences could be explained by distinct basal activation or the differentiation state of the epithelial cells employed [77]. In addition, soluble forms of CD14 from serum plasma can mediate LPS-induced cell activation in epithelial cells under low CD14 membrane expression [79].

NF-*κ*B transcription factor is a key player for cytokine induction; it was stimulated after 4 hrs by ADE organic extracts only from the urban site. More importantly it was shown that this activation was directly related to ENX since PMB was able to significantly reduce NF-*κ*B activation. The IL-8 promoter contains the NF-*κ*B response element [80] indicating that at least part of IL-8 transcriptional activity is dependent on NF-*κ*B as well as IL-6 [81]. Diesel exhaust particles have also been reported to activate this factor in BEAS-2B [48], which demonstrates that human activity can modify the environment activating immunological responses in humans. It appears that PM_10_ is able to promote inflammatory responses through NF-*κ*B. On the contrary, a previous study using PM_2.5_ organic extracts from the same ADE source did not find any NF-*κ*B activation in BEAS-2B [41]. Instead, oxidative stress was found to be activated through Nrf2.

## 5. Conclusions

**Figure 9:**
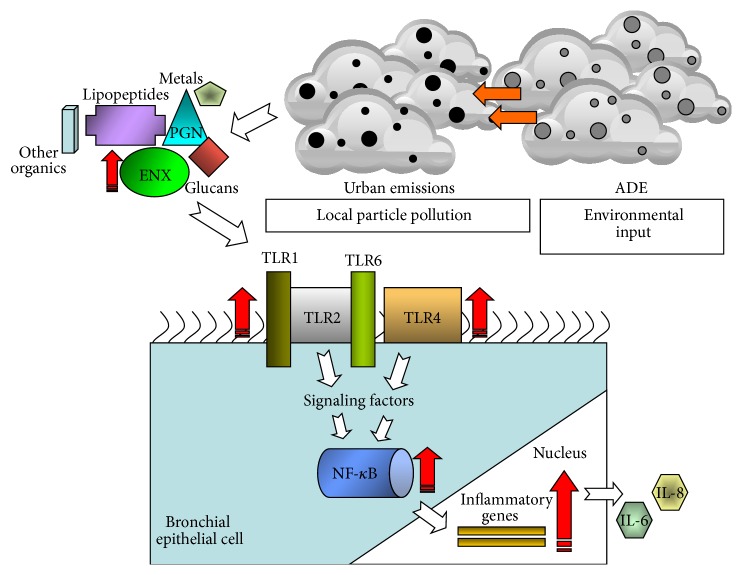
Proposed schematic model of the* in vitro* toxic and inflammatory responses stimulated in the bronchial epithelium. Once African dust (ADE) reaches the urban environment it adds to local particle pollution (increasing PM_10_) augmenting endotoxins and other biological (glucans, peptidoglycans/PGN, and lipopeptides) and chemical constituents (metals and other organics). Biological molecules are recognized by TLRs (TLR4 and TLR2 heterodimerized with TLR1 or TLR6) at the cell surface leading to the activation of signaling pathways and transcription factor, NF-*κ*B which enter the nucleus and induce the expression of proinflammatory genes: IL-6 and IL-8. This effect will aid the bronchial epithelial cells to mediate the microbial respiratory insults. Red arrows and path represent the respective increases found in the present study with ADE at the urban site; and the white arrows and path refer to ADE at the rural site.

This work establishes the importance of differences in bronchial epithelial responses by extracts derived from PM_10_ ADE collected from distinct Puerto Rican areas (urban/Guaynabo versus rural/Fajardo). Particles from different environments differ in their potential to induce inflammatory biomarkers due to different chemical (e.g., organic, inorganic compounds), biological (e.g., ENX), and physical agents (e.g., particle size, seasons, and emissions) [66, 82]. African dust storms can take 3 to 5 days before it reaches the Caribbean [1]. As the dust is transported and settles it becomes mixed with adjacent air masses that contain particular aerosols including biomass-burning products and anthropogenic compounds from various sources [83]. Local urban PM_10_ contains a mixture of microbes and organic and inorganic constituents, which are unique to the site and season. It was noted that African dust reaching PR increases the concentration load of ENX and inorganic and other organic constituents that uniquely interact in urban environments stimulating the secretion of proinflammatory cytokines in targeted cells. By studying African Dust Events using satellite imaging and onsite specific PM_10_ concentrations it was possible to learn more about the duration and occurrence of this global storm phenomenon in Puerto Rico. Furthermore, ADE material at an urban and rural site was successfully obtained to conduct specific* in vitro* testing. The ENX were identified as one of the unique components being incorporated in the local Puerto Rican environment which is biologically active through specific exposure pathways. The ENX interactive complex formed in the environment associates with PM_10_ and strongly activates and induces TLR4 expression while weakly affecting TLR2. This TLR interaction triggers the signal transduction pathway of NF-*κ*B leading to the release of proinflammatory IL-6 and IL-8. The general findings summarizing the present research are presented in Figure 9.

For the first time epidemiological asthma data is directly linked to African dust storms events (March 2004) in Puerto Rico. Using data from a 12-year period, peak of ADE duration was noted at the years of 2002–2005. March and April were the months characterized with greatest storm duration. In addition, a direct correlation between children of 0–5 years suffering from asthma and ADE in the month of March 2004 was reported. Moreover, asthma cases during this period were highly prevalent and amplified at the urban site. The* in vitro* toxicological testing of ADE material from that same month supports these findings.

Using the correct treatments to avoid respiratory and cardiovascular exacerbations during a pollution episode (e.g., ADE) greatly relies on elucidating how and which PM_10_ constituents cause inflammation. The present study contributes in evaluating the effect of ENX derived from African dust reaching Puerto Rico. It points out that adverse health outcomes depend not only on PM mass concentration but also on biological constituents as part of a complex mixture which is modulated by urban emissions. As a matter of public health, these findings greatly recommend preventive measures for the Puerto Rican population since asthma in Puerto Rico is highly prevalent and patients constantly complain of having exacerbations during ADE. More importantly, this research highlights the TLRs as potential targets to consider in the future road of asthma drug therapy and even personalized medicine.

## Figures and Tables

**Table 1:** Correlation between pediatric asthma cases, average PM_10_ concentration, and dust event days in Fajardo and San Juan, Puerto Rico, during 2004 and 2005.

Factors	Unadjusted correlation	
	*R*	*p*-value
Total asthma cases and average PM_10_ concentration		
Fajardo	**0.715**	**0.009**
San Juan	0.139	0.666
Asthma cases (0–5 years) and average PM_10_ concentration		
Fajardo	**0.660**	**0.019**
San Juan	0.178	0.577
Asthma cases (6–12 years) and average PM_10_ concentration		
Fajardo	**0.643**	**0.024**
San Juan	0.081	0.803
Asthma cases (13–18 years) and average PM_10_ concentration		
Fajardo	**0.591**	**0.043**
San Juan	0.096	0.766
Total asthma cases and PM_10_ increment		
Fajardo	**0.687**	**0.013**
San Juan	0.388	0.211
Asthma cases (0–5 years) and PM_10_ increment		
Fajardo	**0.696**	**0.011**
San Juan	0.417	0.177
Asthma cases (6–12 years) and PM_10_ increment		
Fajardo	0.500	0.097
San Juan	0.335	0.286
Asthma cases (13–18 years) and PM_10_ increment		
Fajardo	0.556	0.060
San Juan	0.037	0.910
Asthma cases during non-ADE days		
Fajardo	0.240	0.259
San Juan	0.181	0.409
2004	0.010	0.970
2005	0.036	0.872
Asthma cases during ADE days		
Fajardo	**0.548**	**0.042**
San Juan	0.470	0.089
2004	0.326	0.254
2005	0.369	0.193

Correlation coefficients (*R*) and corresponding *p* values are presented. The closer the *R* is to 1, the stronger is the correlation. Significant *p* values and corresponding *R* are highlighted in bold.

**Table 2:** Endotoxin concentrations in PM_10_ organic extracts from the urban site.

Extracts	EU/mg PM_10_^a^
Non-ADE	116
ADE	168

^a^Endotoxin units per milligram of extracted PM_10_.

## Abbreviations

ENX: Endotoxins
ADE: African Dust Events
Non-ADE: Non-African Dust Events
PMB: Polymyxin B sulfate
TLRs: Toll-like receptors
LPS: Lipopolysaccharide
PGN: Peptidoglycans
